# Supplementary material for: A Metastatic Cancer Expression Generator (MetGen): A Generative Contrastive Learning Framework for Metastatic Cancer Generation
Source: Cancers (Basel). 2024 Apr 25;16(9):1653. doi: 10.3390/cancers16091653 (PMC11083328; doi:10.3390/cancers16091653)
Supplement: Supplementary file 1 [file cancers-16-01653-s001.zip › Supplementary figures.pdf]

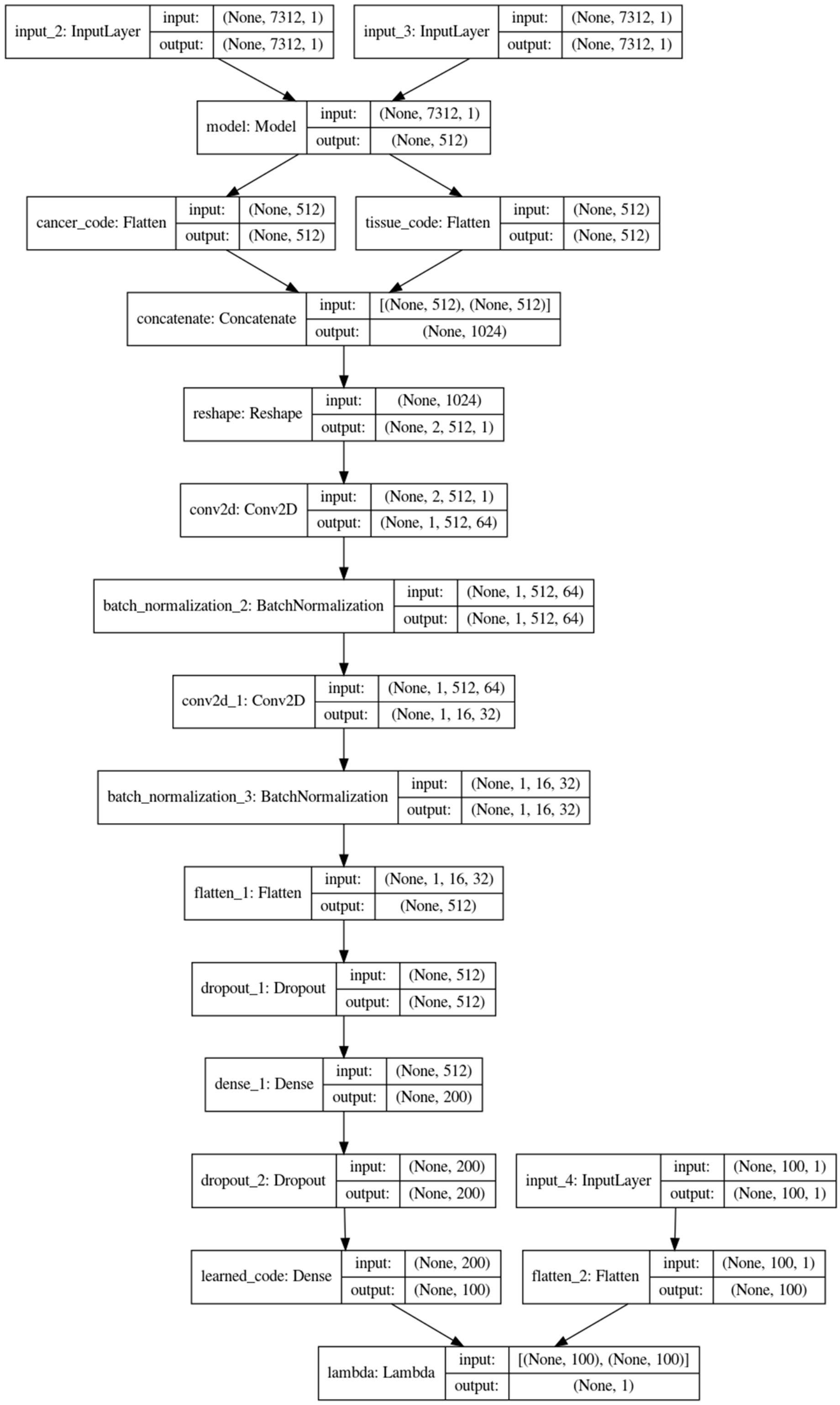

Supplementary Figure S1. Structure of MetGen.

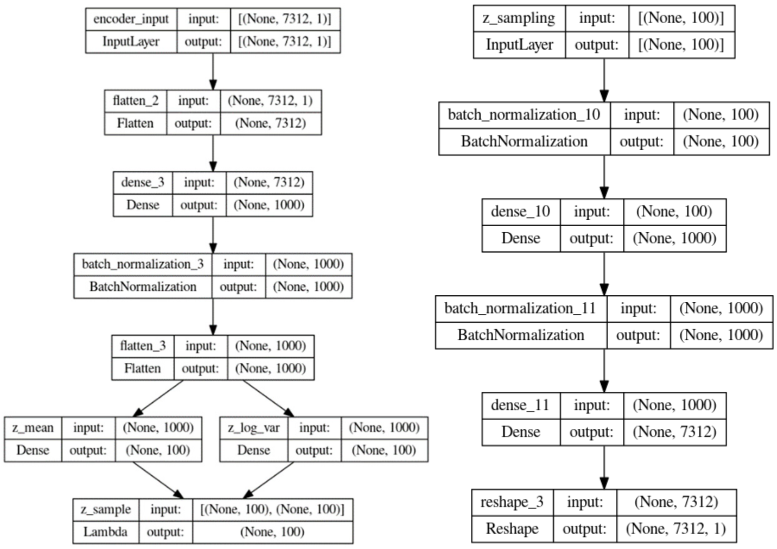

Supplementary Figure S2. Structure of MetVAE.

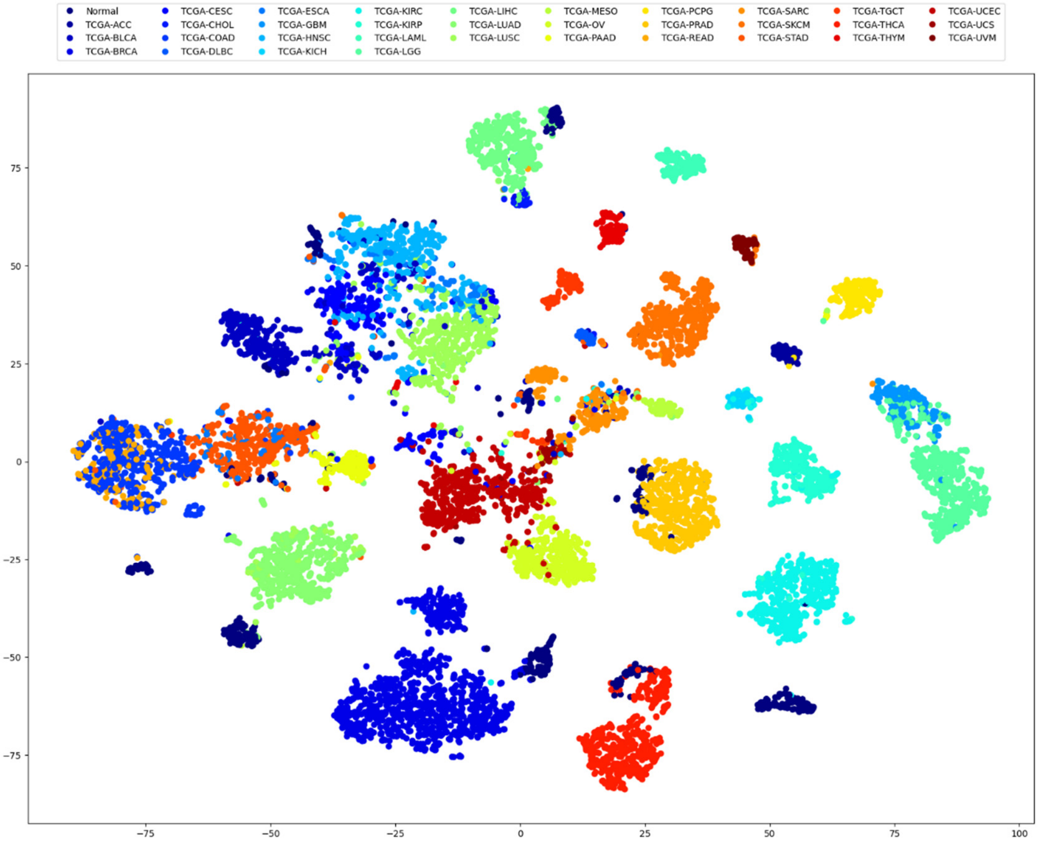

Supplementary Figure S3. Primary tumor distribution.

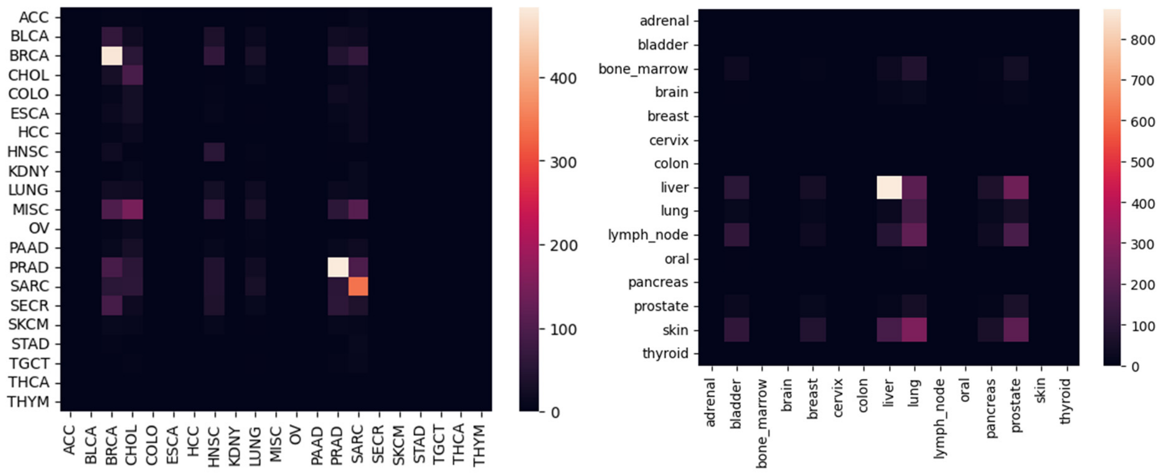

Supplementary Figure S4. Confusion matrix of benchmarking.
